# Space-time couplings in ultrashort lasers with arbitrary nonparaxial focusing

**DOI:** 10.1515/nanoph-2024-0616

**Published:** 2025-01-31

**Authors:** Spencer W. Jolly, Marianna Lytova, Simon Vallières, François Légaré, Steve MacLean, François Fillion-Gourdeau

**Affiliations:** Service OPERA-Photonique, 26659 Université libre de Bruxelles (ULB), Brussels, Belgium; Advanced Laser Light Source (ALLS) at INRS-EMT, 1650 boulevard Lionel-Boulet, Varennes, QC, J3X 1P7, Canada; Infinite Potential Laboratories, Waterloo, ON, N2L 0A9, Canada

**Keywords:** spatio-temporal couplings, tightly focused fields, chromatic aberrations, ultrashort lasers

## Abstract

Space-time separability is commonly assumed in the theoretical description of laser beams. However, recent progresses have demonstrated that this assumption often breaks down for ultrashort realistic pulses, giving rise to spatio-temporal effects that modify both the spatial and temporal characteristics of the laser field. In this work, we introduce semi-analytical and numerical diffraction integral models to investigate these spatio-temporal effects in tightly focused configurations. In particular, we investigate how the TM_01_ beam mode is modified at the focus by chromatic angular dispersion, curvature, and spatial chirp. We compare the two formalisms, thus creating a toolset for modeling extreme localization of structured electromagnetic beams in time and space.

## 1 Introduction

Tightly focused electromagnetic fields have been studied extensively in the last few decades because they are important in many applications such as high-intensity laser science, microscopy, nanophotonics, and many others. Several theoretical approaches have been introduced to describe these intricate field configurations, with varying levels of sophistication and accuracy. Recently, it has become clear that space-time couplings (STCs) [1] also play an important role in the description of these laser beams, particularly when a pulse is short and has a large bandwidth, where STCs can become quite significant [2], [3], [4]. STCs arise from the nonseparability of the laser field into independent time and spatial parts, namely when *E*(*t*, ***x***) ≠ *f*(*t*)*g*(***x***). When this occurs, STCs induce frequency-dependent spatial beam properties in Fourier space.

STCs are especially important for high field science, which generally requires tightly focused short laser pulses to reach the highest levels of intensities and electric field strength. In these circumstances, STCs can be detrimental as they can compromise the pulse duration, alter the pulse shape, or in the worst case scenario, all of the above, resulting in a significant reduction of the intensity at the focus.

On the contrary, one can take advantage of strong STCs because they enable the shaping of laser pulses with a broad bandwidth. As a result, this leads to a notable control over the space-time evolution and behavior of the beam. For example, STCs can be engineered to control high-field laser-matter interactions [5], [6], [7]. Other examples of relevant applications beyond those at high power include laser-based processing of materials and the pumping of nanophotonic, atomic, or molecular systems. For these latter applications, nanometric localization of the electric field is mandatory, which for optical light is only possible with highly nonparaxial focusing.

When it comes to beam control using STCs, previous studies have mostly considered paraxial models for reasons of simplicity and relevance. However, in the tightest focusing geometries, and especially in the case of vectorial laser pulses or field-sensitive phenomena, nonparaxial models are required. Moreover, in high field science, understanding STCs in tightly focused configurations is instrumental to maximize the intensity reached by a given laser system, but these have not been considered extensively. Therefore, this work aims to fill this gap by introducing theoretical descriptions of highly nonparaxial ultrashort laser pulse fields with STCs.

In previous approaches, analytical solutions for the highly nonparaxial case [8], [9] based on the complex source-point model have been investigated. Designed space-time correlations have been added directly in time, resulting in exotic phenomena at the focus [10], [11]. In this article, we will rather address simple and commonplace STCs in a collimated incident beam, subsequently reflected by a highly nonparaxial (high numerical aperture) focusing optic. Our analysis will show how these STCs manifest themselves differently at the focus of a paraxial and highly nonparaxial optic, and how the resultant deformations of the space-time electric field can vary with the numerical aperture. We will first include the STCs corrections in frequency space using a purely analytical model, which requires a frequency-dependent scaling or coordinate transformation, or both. Then, a numerical Fourier transform to view the real fields in time can be performed. We will show some examples of how STCs affect the focus and we will compare them to the paraxial case.

Going beyond the analytical field model and their modifications in the presence of STCs, we will also consider a theoretical framework based on diffraction integrals. The latter has more flexibility because it can model arbitrary intensity or polarization distributions. It is also a more rigorous approach, which can take the actual geometry of the reflector into account and which can reckon with complicated STCs for which there is no known correction to the analytical model. However, it comes with an important computational overhead, which can hinder its use in some applications where the analytical model could outperform the integral model. For these reasons, a comparison will be carried out between the two approaches.

To summarize, in this work, we are building a toolbox for modeling physically realizable STCs in a highly nonparaxial scenario, where there is generally no practical way to assess the nanometric electric field in the focal region. This article is separated as follows. In Section 2, the physical configuration and the modeling approaches are presented. In Section 3, two types of STCs are introduced in the analytical model and numerical results for the fields are obtained. In Section 4, STCs are included in the integral model, and a comparative study is performed with the analytical model. Finally, we conclude in Section 5.

## 2 Physical scenario

### 2.1 The geometry of the problem

**Figure 1:**
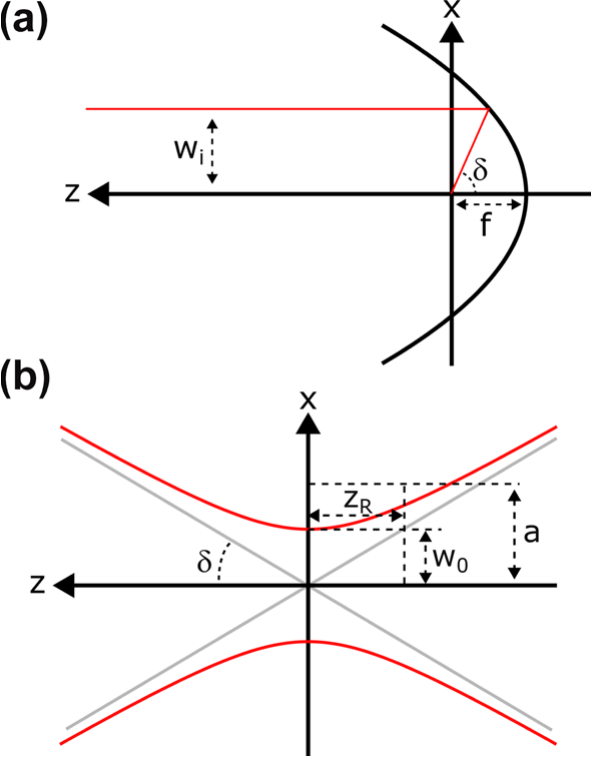
Schematic drawing of the physical setup under consideration. The input parabola is shown in (a), characterized by its focal length *f*, with an input beam (red) having a radius *w*_*i*_ and where the associated half-angle of *δ* is determined from *w*_*i*_ and *f*. The parameters that describe the laser beam in focus are all inter-related, as shown in (b) and detailed in Eqs. (1)–(3), where *δ* makes the connection to the input size and parabola geometry.

The focusing of an optical beam by a high numerical aperture parabolic mirror is shown in Figure 1 both macroscopically in terms of input beam parameters and microscopically around the tight focus. There are of course other possible geometries [12], [13], [14], but we will study this case for its simplicity and widespread use. The most relevant dimension in this configuration is the confocal parameter of oblate spherical coordinates *a*, that is defined most simply in terms of the opening angle of the focusing ray *δ* (NA = sin(*δ*), where NA is the numerical aperture) as

(1) $$a=\frac{2}{k\sin\left(\delta \right)\tan\left(\delta \right)},$$

where *k* = *ω*/*c* with *ω* the angular frequency of the light beam and *c* the speed of light. The more standard quantities of Rayleigh range *z*_*R*_ and beam waist *w*_0_ also have modified definitions:

(2) $$z_{R}=\frac{2}{k\tan^{2}\left(\delta \right)}=\frac{\sqrt{1+{\left(ka\right)}^{2}}-1}{k},$$

(3) $$w_{0}=\frac{2}{k\tan\left(\delta \right)}=\frac{\sqrt{2}\sqrt{\sqrt{1+{\left(ka\right)}^{2}}-1}}{k}.$$

Note that in the paraxial limit *ka* ≫ 1, *z*_*R*_ ≈ *a* and accordingly $w_{0}\approx \sqrt{2z_{R}/k}$ , which are the commonly known paraxial definitions. However, as we will see later, the waist and Rayleigh range as written in Eqs. (2) and (3) do not appear in the analytical field equations and no longer describe the intensity profile accurately [15]. Thus, they should not be interpreted as “physical” parameters as in the paraxial case.

The above relations are only between quantities defined around the focus. However, since most STCs are better defined on the collimated input beam and also usually generated there, we need a way to connect the geometry of the focusing optic to the beam parameters around the focus. Although this connection is straightforward in the paraxial case, we will show here that it is important to consider the actual geometry of the focusing optic in the nonparaxial case.

The surface of the focusing parabola in our physical scenario can be defined using the following equation:

(4) $$z=\frac{r^{2}}{4f}-f,$$

where the focus is fixed at the origin, *f* is the focal length and *r* is the radial distance. Upon inspection, the geometry of the parabola gives

(5) $$\sin\left(\delta \right)={\left(\frac{r}{\sqrt{r^{2}+z^{2}}}\right)}_{r=w_{i}}=\frac{w_{i}}{f+\frac{w_{i}^{2}}{4f}},$$

(6) $$\tan\left(\delta \right)={\left(\frac{r}{-z}\right)}_{r=w_{i}}=\frac{w_{i}}{f-\frac{w_{i}^{2}}{4f}},$$

such that *a* can be calculated in terms of the incoming beam waist and the focal length:

(7) $$a=\left(\frac{2}{k}\right)\left[{\left(\frac{f}{w_{i}}\right)}^{2}-{\left(\frac{w_{i}}{4f}\right)}^{2}\right].$$

This gives us the most important parameter for the field model in terms of the geometry of the input beam and the focusing optic. Also, when the beam becomes paraxial in this context (*w*_*i*_ ≪ *f*), we get *δ* ≈ arctan(*w*_*i*_/*f*) and $a\approx 2f^{2}/kw_{i}^{2}=z_{R}$ , which is as we expect from the paraxial regime.

### 2.2 Modeling directly in time

Now we turn from looking purely at the geometry of the problem, to working with the wave equation. The approach advocated in Refs. [8], [9], [16] is based on exact solutions of the nonparaxial wave equation for Hertz potentials and using the complex source point method [17], [18], [19], [20]. This solution for the scalar potential function *Ψ* is given in frequency space $\tilde{\Psi }$ by

(8) $$\begin{array}{ll} \tilde{\Psi } & ={\tilde{\Psi }}_{0}F\left(\omega \right)\frac{e^{-ka}}{2i}\left[\frac{e^{ik\tilde{R}}}{\tilde{R}}-\frac{e^{-ik\tilde{R}}}{\tilde{R}}\right]\\ & ={\tilde{\Psi }}_{0}F\left(\omega \right)e^{-ka}\frac{\sin\left(k\tilde{R}\right)}{\tilde{R}}, \end{array}$$

(9) $$\tilde{R}=\sqrt{r^{2}+{\left(z+ia\right)}^{2}},\;$$

where ${\tilde{\Psi }}_{0}$ is an overall normalization amplitude. From this solution, the lowest-order fields of many beam modes (TM, TE, LP) can be calculated by choosing the exact vector nature of the potentials. *F*(*ω*) is the spectral amplitude function, which can be a Gaussian (parameterized by the spectral width Δ*ω*) or a Poisson distribution [21] (parameterized by the dimensionless *s*). Depending on the bandwidth and, therefore, pulse duration, we have

(10) $$\begin{array}{cc} F\left(\omega \right) & ={\text{e}}^{\text{i}\psi_{0}}{\left(\frac{s}{\omega_{0}}\right)}^{s+1}\frac{\omega^{s}e^{-s\omega /\omega_{0}}}{\Gamma \left(s+1\right)}\\ & \approx {\text{e}}^{\text{i}\psi_{0}}e^{-{\left(\omega -\omega_{0}\right)}^{2}/\Delta \omega^{2}}\;⟸\;s\gg 10. \end{array}$$

If we assume that there is no hidden frequency dependence in the confocal parameter *a*, and we recognize that *k* = *ω*/*c* is a simple linear function of frequency, then the potential function can be evaluated in the time domain as

(11) $$\Psi =\frac{\Psi_{0}}{\tilde{R}}\left[f\left({\tilde{t}}_{+}\right)-f\left({\tilde{t}}_{-}\right)\right],$$

(12) $${\tilde{t}}_{\pm }=t\pm \tilde{R}/c+ia/c,$$

(13) $$\begin{array}{ll} f\left(t\right) & ={\text{e}}^{\text{i}\psi_{0}}{\left(1-\frac{i\omega_{0}t}{s}\right)}^{-\left(s+1\right)}\\ & \approx {\text{e}}^{\text{i}\psi_{0}}{\text{e}}^{\text{i}\omega_{0}t}e^{-t^{2}/\tau_{0}^{2}}\;⟸\;s\gg 10, \end{array}$$

where *ψ*_0_ is an added carrier envelope phase. Note that *f*(*t*) is the Fourier transform of *F*(*ω*), with *τ*_0_ = 2/Δ*ω* for large *s* (or Gaussian distribution), and that this solution actually contains two parts traveling in opposite directions. This aspect is a reflection of the ideal nature of this model, where the aperture of the focusing optic is actually infinite, and the counter-propagating part of the beam comes from the outer regions of the parabola – if focusing is tighter (*ka* smaller) then the counter-propagating part becomes larger in relative magnitude.

The vectorial part of the potential is now introduced. To stay concise, we consider the most simple case mathematically, which is a purely electric Hertz potential **Π**_*e*_ such that

(14) $$E=\nabla \times \nabla \times \Pi_{e},\;B=\frac{1}{c^{2}}\frac{\partial }{\partial t}\nabla \times \Pi_{e}.$$

The case of the lowest-order radially polarized pulse (TM_01_) is when the potential is $\Pi_{e}=\Psi \hat{z}$ , and cylindrical symmetry allows for the longitudinal field to be calculated using cylindrical coordinate derivatives as follows

(15) $$E_{z}=-\left(\frac{1}{r}\frac{\partial \Psi }{\partial r}+\frac{\partial^{2}\Psi }{\partial r^{2}}\right).$$

Since *Ψ* is formulated as a sum of the temporal function *f*(*t*) but with complex spatio-temporal arguments ${\tilde{t}}_{\pm }$ , the resulting field depends on derivatives of that temporal function as

(16) $$\begin{array}{cc} E_{z} & =\frac{A_{0}}{{\tilde{R}}^{2}}\left[\left(2-\frac{3r^{2}}{{\tilde{R}}^{2}}\right)\left(\frac{f\left({\tilde{t}}_{+}\right)-f\left({\tilde{t}}_{-}\right)}{\tilde{R}}-\frac{f^{\prime }\left({\tilde{t}}_{+}\right)+f^{\prime }\left({\tilde{t}}_{-}\right)}{c}\right)\right.\\ & \left.\;-\frac{r^{2}}{c^{2}\tilde{R}}\left(f^{\prime \prime }\left({\tilde{t}}_{+}\right)-f^{\prime \prime }\left({\tilde{t}}_{-}\right)\right)\right], \end{array}$$

where *f*′ and *f*′′ are the first and second derivatives with respect to time of the temporal function, respectively. The other nonzero field components are given in Appendix A.

In the paraxial equation, specific space-time correlations and so-called time-diffraction have been shown by taking advantage of the duality between space and time in the wave equation [22], [23], [24], [25], [26], [27]. It is an interesting question how space-time structures can be built on the highly nonparaxial pulses derived in this section using an ansatz inspired by time-diffracting beams, as done in previous work [10], [11], [28]. However, as described in the introduction, we focus in this work on more simple and, therefore, experimentally relevant space-time effects. To do this, we first show modeling of the potentials and fields in frequency space, where the STCs can be more easily added.

### 2.3 Modeling in frequency space

The nonparaxial model from Refs. [8], [9], [16] uses Hertz potentials as solutions of the nonparaxial wave equation, which were outlined in the previous section in the temporal domain. The electric and magnetic fields can rather be formulated in frequency space, beginning with the same equation for the potential (Eq. (8)), but making no assumptions on the frequency dependence of *a* (and therefore $\tilde{R}$ ). We consider again the most simple case mathematically, which is a purely electric Hertz potential ${\tilde{\Pi }}_{e}$ but expressed in frequency space:

(17) $$\tilde{E}=\nabla \times \nabla \times {\tilde{\Pi }}_{e},\;\tilde{B}=\frac{i\omega }{c^{2}}\nabla \times {\tilde{\Pi }}_{e}.$$

For the lowest-order radially polarized pulse (TM_01_), the potential is given by ${\tilde{\Pi }}_{e}=\tilde{\Psi }\hat{z}$ ; therefore, the longitudinal field can be calculated in the same way as was done directly in time in the previous section, but now using the frequency domain potential $\tilde{\Psi }$ . This yields

(18) $$\begin{array}{cc} {\tilde{E}}_{z} & =\frac{{\tilde{A}}_{0}F\left(\omega \right)e^{-ka}}{{\tilde{R}}^{2}}\left[\sin\left(k\tilde{R}\right)\left(\frac{2+k^{2}r^{2}}{\tilde{R}}-\frac{3r^{2}}{{\tilde{R}}^{3}}\right)\right.\\ & \;+\left.\cos\left(k\tilde{R}\right)\left(\frac{3kr^{2}}{{\tilde{R}}^{2}}-2k\right)\right]. \end{array}$$

The other field components are written in Appendix B. The amplitude term required to have physical units for the field is given by

(19) $${\tilde{A}}_{0}=\sqrt{\frac{8P}{\pi \epsilon_{0}c}}\frac{f\left(a,s\right)}{2k_{0}}.$$

Importantly, the function *f*(*a*, *s*) is left undefined for the moment, although there exist certain solutions [29]. It is known, for example, that *f*(*a*, *s*) ∼ *a*/Δ*ω* in the paraxial limit (*k*_0_ *a* ≫ 1) and with a Gaussian spectral envelope (*s* ≫ 10).

Past work have focused on the power in such a beam [29]. It was concluded that the power when *a* = 0 can be zero, since an equal amount of energy is flowing in the +*z* and −*z* directions. However, energy must be conserved because we are dealing with a physical input pulse with a finite power and total energy. How to properly define *f*(*a*, *s*) is, therefore, a work in progress and is outside the scope of this article. To avoid complicating the calculation of this function, we will not consider a frequency-dependent confocal parameter *a*(*ω*).

If *a* is assumed to be frequency-independent at this point, then the potential or fields calculated here can be Fourier transformed to find the potential or fields in time for any polarization [9]. Directly Fourier-transforming the longitudinal field solution shown here in the case of a TM_01_ beam, Eq. (18), agrees with Eq. (16) (the two approaches are equivalent). This can be seen in comparing the two equations and noticing that a term ∝ *k* ^*n*^ *F*(*ω*) will be related to the *n*-th derivative of *f*(*t*). However, if the situation is more general by having *a*(*ω*) (not considered in this work) or other imposed space-time couplings, then the vector potential or the fields in time must be calculated numerically. In Section 3, we will first discuss the implications and scalings of different space-time effects on this model before revisiting the solutions for the fields including the added STCs.

### 2.4 Model based on vector diffraction integrals

The analytical field model presented in the previous section is powerful as it allows for describing the fields in the focus with a low computational cost. However, the analytical field model has several limitations, even when the spatio-temporal distortions are included. In particular, it can only accommodate for lowest-order Gaussian transverse distributions (whether LP, TE, or TM), it does not include any high-spatial-frequency or high-order phase aberrations, it assumes an infinite aperture of the focusing parabola, and in general cannot handle any other complexities.

Integral methods, generally based on the Stratton–Chu [30] or Richards–Wolf [31] diffraction integrals, provide a significantly more complete description [32], but they are more computationally expensive. Assuming the reflector is a perfect conductor and using the physical optics approximation (where the radius of curvature of the reflecting surface R is much larger than the wavelength, *i.e.* R≫λ [33]), the Stratton–Chu integral equations for a spectral component at frequency *ω*_*n*_ are given by [34]:

(20) $$\begin{array}{ll} E_{n}\left(x\right) & =\frac{1}{2\pi }\underset{S}{\int }\left\{ik_{n}\left(\hat{N}\times B^{\text{inc}}\right)G_{n}+\left(\hat{N}\cdot E^{\text{inc}}\right)\nabla_{S}G_{n}\right\}dS, \end{array}$$

(21) $$\begin{array}{ll} B_{n}\left(x\right) & =\frac{1}{2\pi }\underset{S}{\int }\left\{\left(\hat{N}\times B^{\text{inc}}\right)\times \nabla_{S}G_{n}\right\}dS, \end{array}$$

where S is the surface of the mirror, ***E*** ^inc^ and ***B*** ^inc^ is the incident electromagnetic field on the surface, dS is a mirror section, $\hat{N}$ is the unit vector normal to the mirror and *k*_*n*_ is the wave vector of the *n*th spectral component. Moreover, *G*_*n*_ is the Green’s function given by:

(22) $$G_{n}\left(x,x_{S}\right)=\frac{e^{ik_{n}|x-x_{S}|}}{\left|x-x_{S}\right|},$$

where $x_{S}$ are coordinates confined to the surface S . To represent short pulses, many spectral components are evaluated and coherently summed as

(23) $$E\left(t,x\right)=2\overset{N_{s}}{\underset{n=1}{\sum }}Re\left[E_{n}\left(x\right){\text{e}}^{-\text{i}\omega_{n}t}\right],$$

(24) $$B\left(t,x\right)=2\overset{N_{s}}{\underset{n=1}{\sum }}Re\left[B_{n}\left(x\right){\text{e}}^{-\text{i}\omega_{n}t}\right],$$

where *N*_*s*_ is the number of spectral component (the *n*’th spectral angular frequency is given by *ω*_*n*_ = *n*Δ*ω*). The computational complexity to perform this calculation numerically is $O\left(N_{s}N_{S}N_{c}\right)$ , where $N_{S}$ is the number of quadrature points on the surface and *N*_*c*_ is the number of points where the field is calculated. The numerical evaluation of the surface integral incurs an important overhead compared to the analytical model, for which the computational complexity scales like *O*(*N*_*s*_ *N*_*c*_). There exist many possible avenues to improve the performance of the integral model. The simplest one would be to consider a fast Fourier transform, when *N*_*s*_ = *N*_*c*_, which would lead to a computational complexity of $O\left(N_{S}N_{s}\ln N_{s}\right)$ . Other ideas are presently under investigation, like using neural networks techniques or preintegrating the frequencies analytically.

Using the flexibility of the diffraction integral method, many applications of this framework have been considered. For example, it has been shown that tuning the transverse distribution or the aperture for radially polarized light significantly affects the Gouy phase [35], [36], [37]. The focusing characteristics of different kinds of parabolic reflectors using linearly and radially polarized beam have been investigated [13]. Exotic spatio-temporal beams have also been studied [38], [39], [40]. In this work, as presented in Section 4, we will rather explore the manifestation of simple couplings in the tight-focusing geometry [41] and especially compare to the analytical model.

## 3 Adding chromatic terms to the analytical model

In this section, we will essentially discuss how to model a number of STCs using the analytical model in frequency space (Section 2.3). We will then show how to implement those constructed models in the vector potential, and finally show results.

### 3.1 Input with angular dispersion

**Figure 2:**
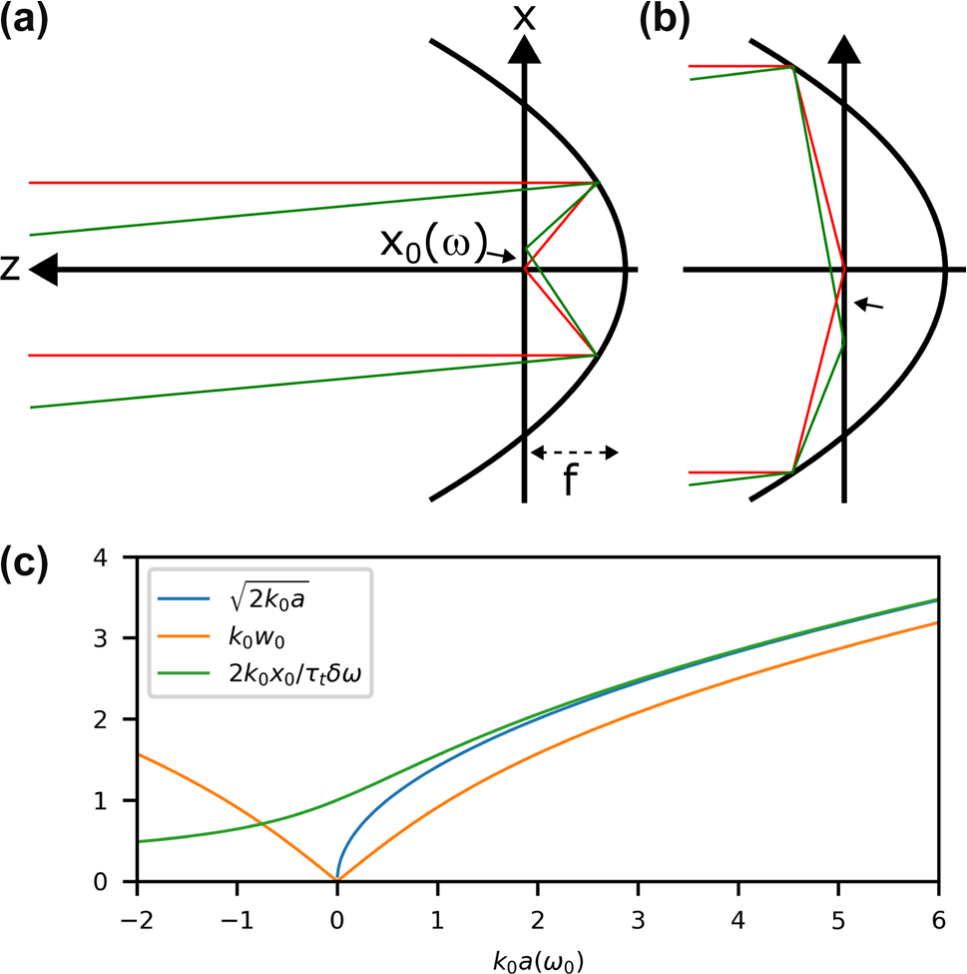
Input with angular dispersion. (a) Schematic of angular dispersion on the input leading to spatial chirp in the focus, seen in the transverse offset of the focus (green) when compared to the central frequency that has no input tilt (red). A limitation in this case is that counter-propagating rays (b) have an offset of a different sign. Different length scales (c) are shown, emphasizing how the correct relationship for *x*_0_(*ω*) (green) differs from those using $\sqrt{a}$ (blue) or *w*_0_ (orange) as the scaling. The three values become similar in the paraxial limit (*ka* ≫ 1).

When angular dispersion is present in one direction on the input pulse, it results in a frequency-dependent offset along that same Cartesian coordinate, as seen in the sketch of Figure 2a. We refer to this phenomenon as *spatial chirp* (SC). If we take *x* to be the relevant Cartesian axis, we must perform the operation *x* → *x* − *x*_0_(*ω*) to model the SC in the focus, which gives

(25) $${\tilde{R}}_{\text{SC}}\left(\omega \right)=\sqrt{{\left[x-x_{0}\left(\omega \right)\right]}^{2}+y^{2}+{\left[z+ia\right]}^{2}}.$$

Then, the electromagnetic potential in Eq. (8) is modified with the substitution $\tilde{R}\to {\tilde{R}}_{\text{SC}}$ .

A strong limitation of this phenomenological model is that rays corresponding to the counter-propagating part of the solution, seen in Figure 2b, will actually result in an offset of negative sign. Furthermore, when we insert this in the equations for the potential, it results in a singularity (i.e., nonphysical value) at *z* = 0. We will attempt to clarify the importance of these features when comparing to the integral models.

The parabolic mirror, which we impose as the geometry of this scenario, also changes the relationship of the angular dispersion magnitude before the parabola and the SC at the focus. The angular dispersion existing on the pulse input of the parabola is equivalent to a pulse-front tilt *τ*_*t*_ (the group delay on the input beam at *r* = *w*_*i*_ is *τ*_*t*_). This induces a frequency-dependent transverse waist position (spatial chirp) in the focus of the form

(26) $$x_{0}\left(\omega \right)=\frac{f\tau_{t}\delta \omega }{kw_{i}},$$

where *δω* = *ω* − *ω*_0_, i.e., the frequency difference from the central frequency *ω*_0_.

In the paraxial case, these quantities could easily be related to physical parameters in the sense that *x*_0_ could be written in terms of the beam waist or Rayleigh length. In the highly nonparaxial case, this can no longer be performed. Past work using numerical simulations showed that the offset in the focus is still linearly dependent on the angle [42], so we can continue with that assumption. However, we need to find an expression for the *x*_0_ using the confocal parameter. Using Eq. (7), we can find that

(27) $$\frac{f}{w_{i}}=\frac{1}{2}\sqrt{\sqrt{1+{\left(ka\right)}^{2}}+ka},$$

such that

(28) $$x_{0}\left(\omega \right)=\sqrt{\sqrt{1+{\left(ka\right)}^{2}}+ka}\frac{\tau_{t}\delta \omega }{2k}.$$

Only in the paraxial approximation does *x*_0_ ≈ *w*_0_ *τ*_*t*_ *δω*/2, which is the relation used in previous work and a simplification of Eq. (28). Different possible scalings are shown in Figure 2c where it is clear that as *ka* becomes small it is important to use the correct relation in Eq. (28). Note finally that *k* = *ω*/*c* remains in Eq. (28), which adds an additional weak frequency dependence not easily visualized in Figure 2 and that only becomes non-negligible when the pulse is approaching single-cycle duration.

The lateral shift is the strongest effect of the input angular dispersion (frequency-dependent tilt). One can also see in the exaggerated schematic in Figure 2 that there would also be an angle difference. We implicitly assume that *x*_0_(*ω*) is small enough such that this angular dispersion in the focus can be neglected and will rather treat angular dispersion as a separate space-time effect in a later section. There are other effects of tilted input on a high-NA parabola, such as coma (which would be frequency-dependent in this case) [42], that cannot be modeled using the simple phenomenology of this section. This will be an aspect of the comparison to integral methods in a later section, which can account for such complexities.

### 3.2 Input with chromatic curvature

**Figure 3:**
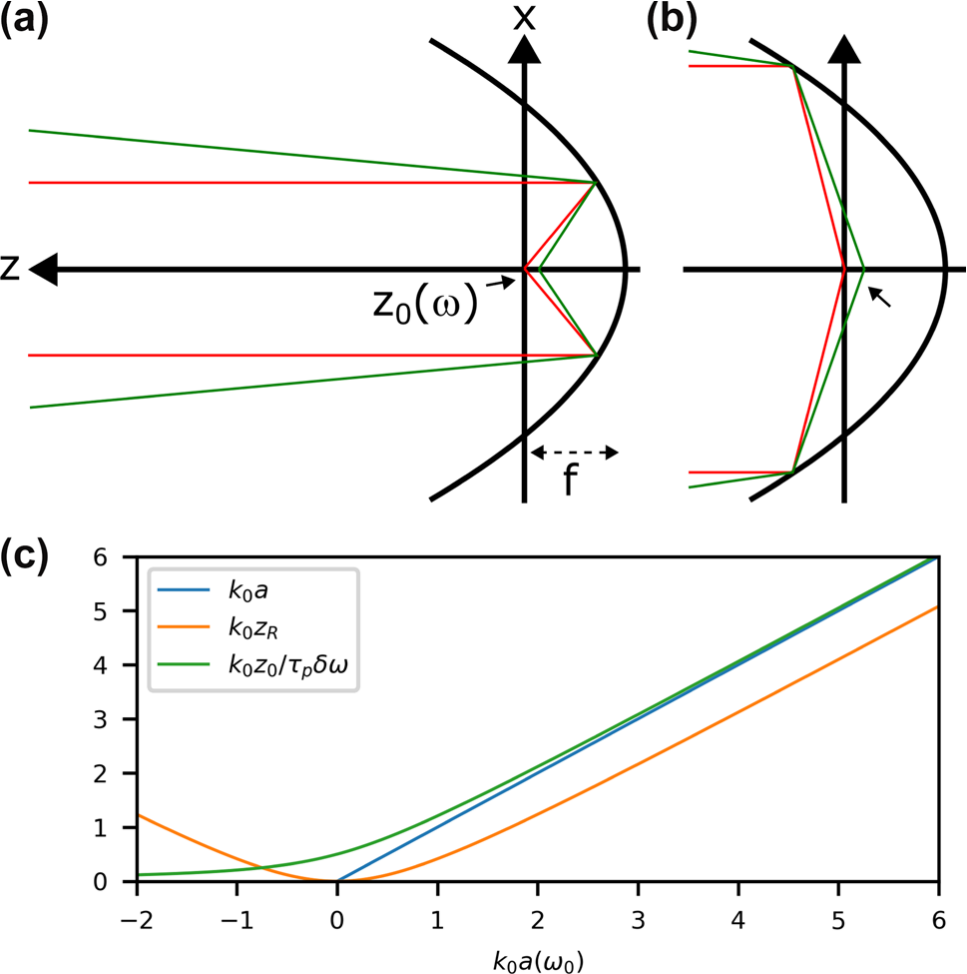
Input with chromatic curvature. (a) Schematic of chromatic curvature on the input leading to longitudinal chromatism in the focus, seen when comparing the focal position for a noncentral frequency (green) when compared to the central frequency (red). Rays that are counter-propagating (b) have the same chromatism. Different length scales (c) are shown, emphasizing how the correct relationship for *z*_0_(*ω*) (green) differs from both *a* (blue) and *z*_*R*_ (orange). All three values become similar in the paraxial limit (*ka* ≫ 1).

When chromatic curvature is present on the input pulse, it will generate a frequency-dependent offset along the longitudinal coordinate, as displayed in the sketch of Figure 3a, which we refer to as *longitudinal chromatism* (LC). To model this LC in the focus, we must perform the operation *z* → *z* − *z*_0_(*ω*) in the field model, which gives

(29) $${\tilde{R}}_{\text{LC}}\left(\omega \right)=\sqrt{x^{2}+y^{2}+{\left[z-z_{0}\left(\omega \right)+ia\right]}^{2}}.$$

Then, the electromagnetic potential is modified by performing the substitution $\tilde{R}\to {\tilde{R}}_{\text{LC}}$ . One can see in Figure 3b that rays corresponding to the counter-propagating part of the solution have the same *z*_0_(*ω*), which means that there is no issue in this regard.

A chromatically varying phase curvature (defocus) of the beam input on the focusing optic is equivalent to a pulse-front curvature *τ*_*p*_ (the group delay on the input beam at *r* = *w*_*i*_ is *τ*_*p*_). Following the derivation in Ref. [43], it results in the following:

(30) $$z_{0}\left(\omega \right)=\frac{2f^{2}\tau_{p}\delta \omega }{kw_{i}^{2}}.$$

At first sight, this equation depends on the input beam waist and the focal length of the focusing optic and not local properties around the focal point. However, using once again Eq. (27), we find

(31) $$z_{0}\left(\omega \right)=\left(\sqrt{1+{\left(ka\right)}^{2}}+ka\right)\frac{\tau_{p}\delta \omega }{2k}.$$

Only in the paraxial approximation does *z*_0_ ≈ *z*_*R*_ *τ*_*p*_ *δω*, which is the relation used in previous work. Different possible scalings are shown in Figure 3c where it is clear that as *k*_0_ *a* becomes small it is important to use the correct relation in Eq. (31). Once again, since *k* = *ω*/*c* is explicitly in Eq. (31), there is an additional frequency dependence that becomes important when the pulse reaches single-cycle duration, that is not easily visualized in Figure 3.

The coordinate change *z* → *z* − *z*_0_(*ω*) is essentially giving each frequency a different complex source point. However, with different source points, the colors would have different phases at any given plane. When considering a pulse with chromatic curvature focused with a parabola, there should not be any difference in constant phase between the frequencies at any given plane (only a different curvature). This means that an additional phase correction of exp(−*ikz*_0_(*ω*)) is needed to completely bridge the gap between the input collimated beam and the model around focus. This will be confirmed later.

### 3.3 Input with spatial chirp

**Figure 4:**
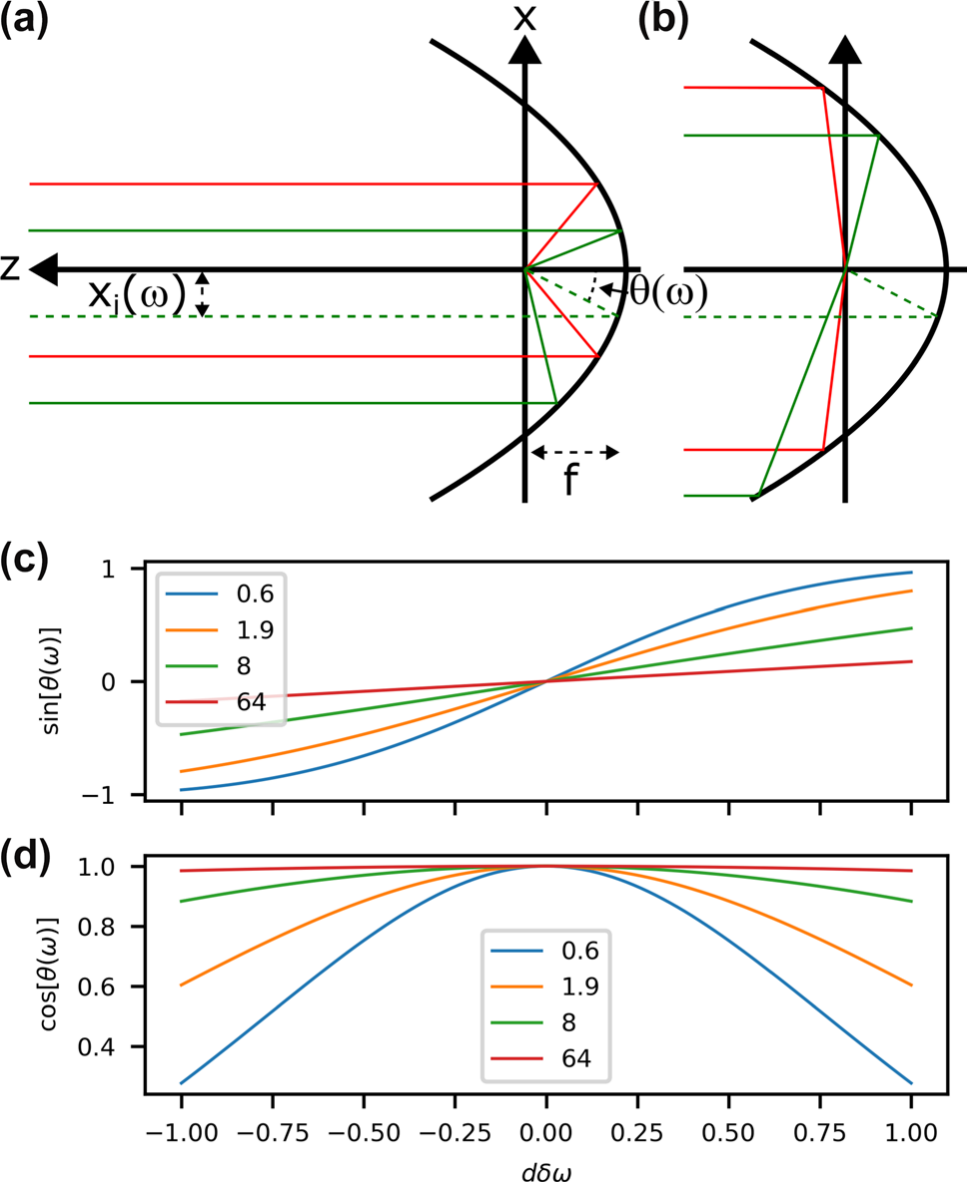
Input with spatial chirp. (a) Schematic of a single-frequency (green) offset in *x* by *x*_*i*_ resulting in an angle of propagation *θ* compared to the central frequency (red) that has no offset or angle. Counter-propagating rays (b) also have this angle offset, but asymmetry is introduced at the extremes. The resulting angle *θ*(*ω*) (c–d) has a nonlinear dependence for highly nonparaxial focusing (small *ka*, see legends), which can be seen in sin[*θ*(*ω*)] (c) deviating from a linear dependence and cos[*θ*(*ω*)] (d) deviating from 1.

If the input pulse has a spatial chirp, there will be an *angular dispersion* (AD) at the focus, as shown in Figure 4a. If the input spatial chirp is *x*_*i*_(*ω*), then there is a frequency-dependent angle *θ*(*ω*). This results in each frequency being in its own rotated coordinate space {*x*′, *y*, *z*′} according to *θ*(*ω*), where

(32) $$x^{\prime }=x\cos\left[\theta \left(\omega \right)\right]-z\sin\left[\theta \left(\omega \right)\right],$$

(33) $$z^{\prime }=z\cos\left[\theta \left(\omega \right)\right]+x\sin\left[\theta \left(\omega \right)\right].$$

To solve for the fields, we have to operate in one common coordinate space {*x*, *y*, *z*}. Therefore, this results in

(34) $$\begin{array}{cc} {\tilde{R}}_{\text{AD}}\left(\omega \right) & =\sqrt{{\left(x^{\prime }\right)}^{2}+y^{2}+{\left[z^{\prime }+ia\right]}^{2}}\\ & =\sqrt{{\tilde{R}}^{2}+2ia\left(z\left(\cos\left[\theta \left(\omega \right)\right]-1\right)+x\sin\left[\theta \left(\omega \right)\right]\right)} \end{array}$$

for the relevant variables within the potential. In addition, we must do a coordinate change for the potential itself due to the modification in propagation direction, which will be discussed later. Usually in the paraxial case, the vector nature of light is not taken into account for angular dispersion and vectorial components do not mix. However, we must allow for this in the nonparaxial case, which is achieved by performing the coordinate change of the vector orientation of the potential.

The rationale that we have used so far applies also to rays corresponding to the counter-propagating part of the potential, as displayed in Figure 4b. At these extremes, an asymmetry may be introduced in the amplitude of the counter-propagating part of the solution, which cannot be modeled in our case since it would produce a singularity at *z* = 0.

Just like the other STCs, scaling is also important here. In the paraxial regime, *θ*(*ω*) could be assumed to be small such that sin[*θ*(*ω*)] ∼ *x*_*i*_(*ω*)/*f* and cos[*θ*(*ω*)] ∼ 1. However, in the strongly nonparaxial case, this is not true. We must use the same calculations for Eqs. (5) and (6) and the relationships between *a* and the focusing parameters in Eq. (7) and its inverse Eq. (27). Finally defining *x*_*i*_(*ω*) = d*w*_*i*_ *δω*, we find

(35) $$\begin{array}{l} \begin{array}{cc} \sin\left[\theta \left(\omega \right)\right] & =\frac{x_{i}\left(\omega \right)}{f+\frac{{\left[x_{i}\left(\omega \right)\right]}^{2}}{4f}}\\ & =\frac{2d\delta \omega \sqrt{\sqrt{1+{\left(ka\right)}^{2}}+ka}}{ka+\sqrt{1+{\left(ka\right)}^{2}}+{\left(d\delta \omega \right)}^{2}}, \end{array} \end{array}$$

(36) $$\begin{array}{l} \begin{array}{cc} \cos\left[\theta \left(\omega \right)\right] & =\frac{f-\frac{{\left[x_{i}\left(\omega \right)\right]}^{2}}{4f}}{f+\frac{{\left[x_{i}\left(\omega \right)\right]}^{2}}{4f}}\\ & =\frac{ka+\sqrt{1+{\left(ka\right)}^{2}}-{\left(d\delta \omega \right)}^{2}}{ka+\sqrt{1+{\left(ka\right)}^{2}}+{\left(d\delta \omega \right)}^{2}}. \end{array} \end{array}$$

These nonparaxial relations lead to a nontrivial coordinate transformation in the modified field model that is nonlinear in *δω*, visualized in Figs. 4c and d. Furthermore, if *x*_*i*_(*ω*) ≪ *f* (*dδω* ≪ *ka*) for all relevant frequencies (dΔ*ω* ≪ *ka* in the Gaussian spectrum case), then the nonlinearity in frequency for sin[*θ*(*ω*)] is no longer present and cos[*θ*(*ω*)] ∼ 1. If additionally *k*_0_ *a* ≫ 1 (paraxial), then sin[*θ*(*ω*)] ∼d*w*_*i*_ *δω*/*f* and we recover the paraxial result as one would expect. This is seen in Figs. 4c and d, where the paraxial relations are true for all d*δω* shown if *ka* is large, or only true for a small range of d*δω* when *ka* becomes small. It is known in the paraxial case, and it will also be confirmed later, that a SC of the input beam results in a pulse-front tilt in the focus corresponding to an AD. Arbitrary nonparaxial pulses with a pulse-front tilt have been derived before [11], but those solutions differ in that the spectral function *F*(*ω*) also has space-frequency dependence, whereas in our case we consider a pure coordinate change.

As a final note, for propagation sufficiently far away from the focus, a pulse with angular dispersion will eventually develop spatial chirp and a group-delay dispersion (temporal chirp). However, it is often the case that to generate the spatial chirp on the input beam a global temporal chirp is also imparted, such that when focused, temporal chirp is no longer present in the focus. This is essentially the simultaneous spatial and temporal focusing (SSTF) method [44] that has shown promise for laser-based processing [45], [46], [47]. Therefore, since we are constructing our model in the focal region, we will not consider any group-delay dispersion.

### 3.4 The effect on the analytical field model

For completion, we now combine the results for the three STCs considered and described in previous subsections. The analytical field model presented in Section 2.3 must be modified according to the longitudinal and transverse frequency-dependent shifts *z*_0_(*ω*) and *x*_0_(*ω*), respectively, and the coordinate transformation due to angular dispersion parametrized by *θ*(*ω*). This requires the updated quantities

(37) $$r_{\text{STC}}=\sqrt{{\left(x-x_{0}\right)}^{2}+y^{2}},\;$$

(38) $${\tilde{R}}_{\text{STC}}=\sqrt{\begin{array}{cc} & r_{\text{STC}}^{2}+{\left[z-z_{0}+ia\right]}^{2}\\ & +2ia\left[\left(z-z_{0}\right)\left(c_{\theta }-1\right)+\left(x-x_{0}\right)s_{\theta }\right] \end{array}},$$

where *c*_*θ*_ and *s*_*θ*_ are shorthand for cos[*θ*(*ω*)] and sin[*θ*(*ω*)], respectively, and the implicit frequency dependence is not shown for all of *x*_0_, *z*_0_, *c*_*θ*_, and *s*_*θ*_ to save space.

However, this so far only includes spatial coordinate transformations and not the vector nature of the potential. Particularly for AD, a further coordinate change is necessary on vector components. In general, this requires changing whichever vectors are relevant. For the TM_01_ beam where ${\tilde{\Pi }}_{e}=\tilde{\Psi }\hat{z}$ when there are no STCs, which was studied earlier and is the simplest potential, the vector potential with all STCs discussed is as follows

(39) $$\begin{array}{cc} {\tilde{\Pi }}_{e}= & {\tilde{\Psi }}_{\text{STC}}\left(x^{\prime }-x_{0}\left(\omega \right),y,z^{\prime }-z_{0}\left(\omega \right),\omega \right){\hat{z}}^{\prime }\\ = & {\tilde{\Psi }}_{0}F\left(\omega \right)e^{-ka}e^{-ikz_{0}\left(\omega \right)}\frac{\sin\left(k{\tilde{R}}_{\text{STC}}\right)}{{\tilde{R}}_{\text{STC}}}\\ & \times \left(\cos\left[\theta \left(\omega \right)\right]\hat{z}+\sin\left[\theta \left(\omega \right)\right]\hat{x}\right). \end{array}$$

This equation is the most important analytical result of this section. The term exp(−*ikz*_0_(*ω*)) is required to compensate for the phase added in the focus from artificially inserting *z*_0_(*ω*). We know that the input beam on the parabola has all colors in phase and thus, they must be in phase with each other in the focus as well. Since we are adding a *real* source displacement along the direction of propagation within $\tilde{R}$ , and therefore adding a phase, we have to compensate for it with the additional term. In other words, we know *a priori* that the beam is not originating from the real source points and rather arriving there from a focused collimated beam where all frequencies are in phase.

The fields from the space-time potential can be calculated using Eq. (17) in Cartesian form. As an example, some field components are presented when there is no angular dispersion in Appendix C.

In the next section, we will generally consider only one central wavelength and only one pulse duration, while focusing levels and strengths of the different STCs will be varied. However, the results can be extended readily to other parameter values. Due to the implicit increase of the dimensionless *ka* = *ωa*/*c*, decreasing the central wavelength will simply make the beam smaller for a given focusing strength, which is defined by the confocal parameter *a*. Furthermore, when the pulse duration is reduced, the effect of a given STC will be stronger for the same magnitude of the parameter (seen in the linear dependence on *δω* in all STC terms) – we study similar effects when we keep the duration constant but increase the strength of the STCs in the following sections. Indeed, as the focusing gets very tight *ka* → 0 or the duration very small *s* ∼ 1, there may be interesting nuances and unforeseen limits to the model. For these reasons, we purposefully avoid these extreme cases, which could be investigated in future studies on the same topic.

### 3.5 Numerical results

Numerical results for the spatial and temporal dependence of the fields are now presented for AD, SC, and LC. For all results, we choose a wavelength of 800 nm, which is typical for high-intensity laser beams.

#### 3.5.1 Input with spatial chirp

**Figure 5:**
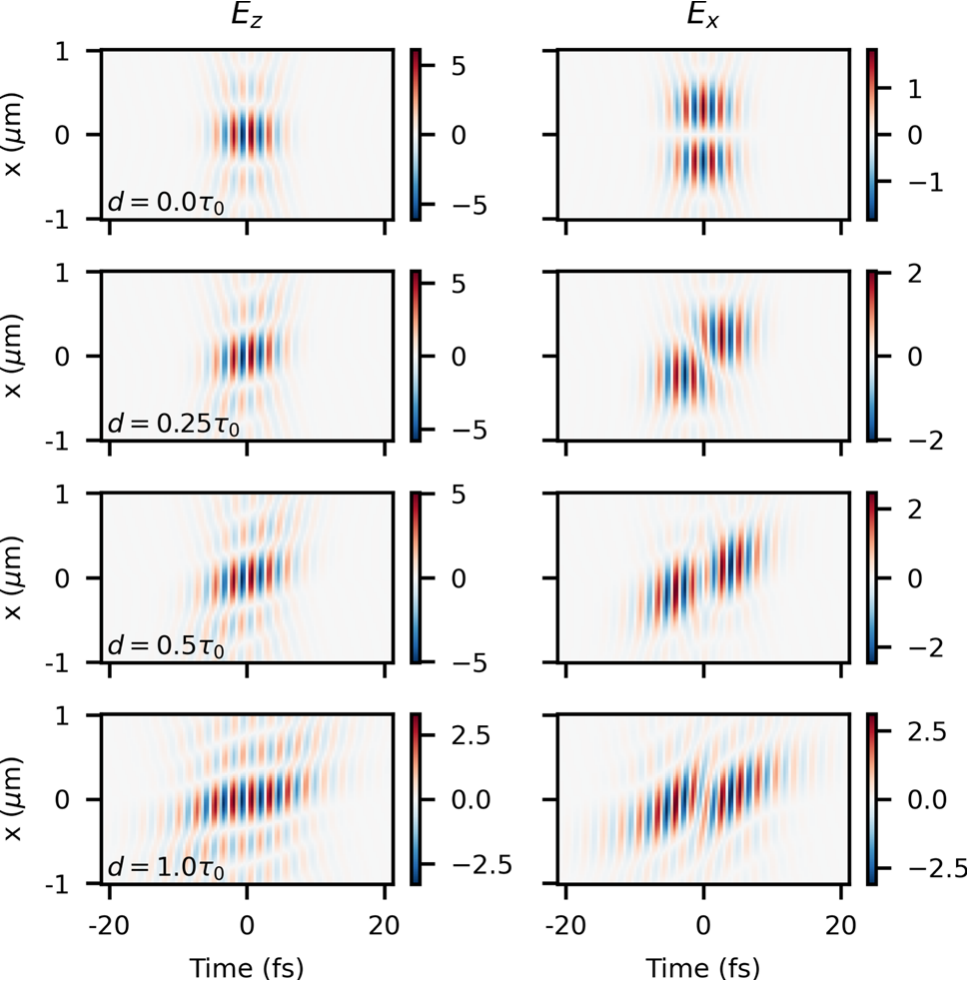
The electric field for both *E*_*z*_ (left) and *E*_*x*_ (right) at *y* = *z* = 0 for an 800 nm pulse of *s* = 50 (duration *τ*_0_ = 4.25 fs) at an NA of 0.8 with angular dispersion parameterized by *d* = *τ*_0_ × {0.0, 0.25, 0.5, 1.0} (from top to bottom).

Results at the focus (*z* = 0) for NA = 0.8 (*k*_0_ *a* = 1.9) can be seen in Figure 5 for four different values of angular dispersion, parametrized by *d*. At such a high NA, the spot is on the submicron scale, with the longitudinal field stronger than the transverse field – seen clearly in the case without any angular dispersion (*d* = 0). As the angular dispersion increases in magnitude, both field components develop a pulse-front tilt as we expect, although the tilt in the longitudinal field is different from the transverse field, as was already noted in previous paraxial results [48]. However, at higher values of angular dispersion, both field components become significantly warped in time and space, and warped in a different manner. This is clearly due to the combination of effects of the nonlinearity of the angular dependence on frequency as evidenced in Eqs. (35) and (36) and the mixing of the vectorial components of the potential in Eq. (39). Separating the effects of both sources of distortion is difficult, but regardless these results show that the nonparaxial case is significantly more complicated than the paraxial case, and that established phenomenon, like SSTF [44], may not be as viable in highly nonparaxial scenarios.

**Figure 6:**
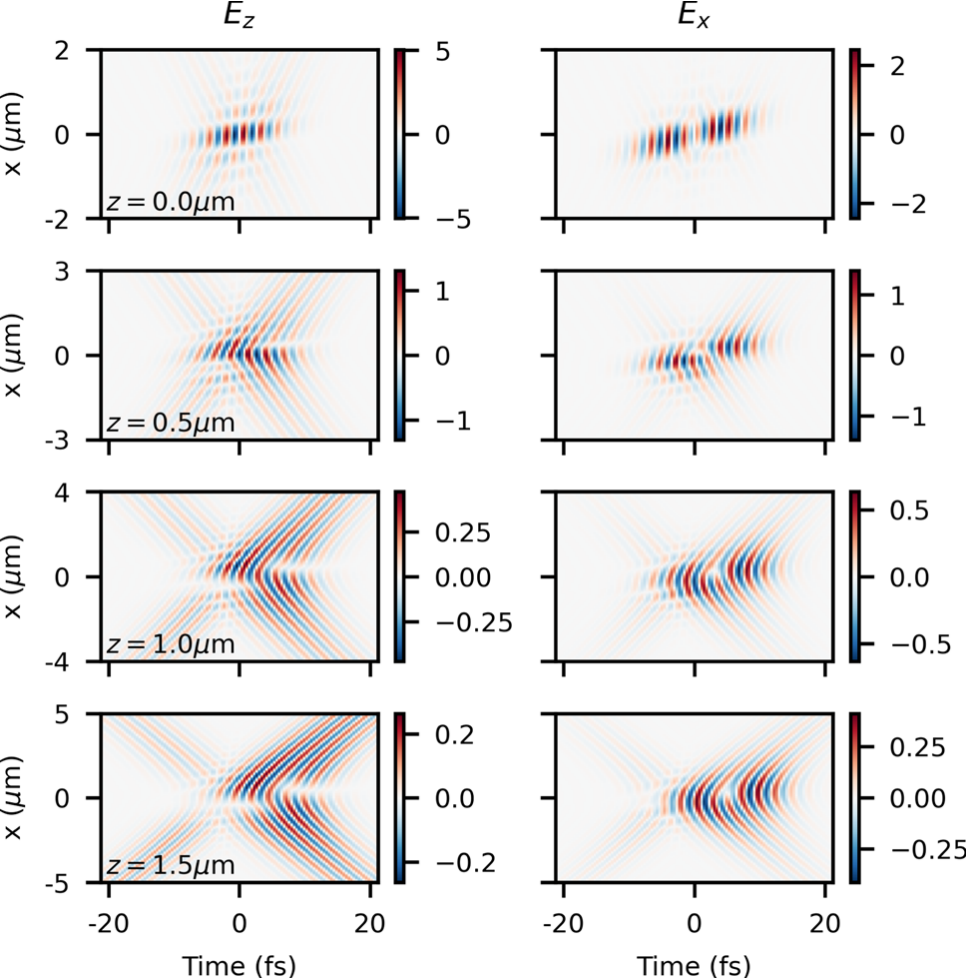
The electric field on propagation for both *E*_*z*_ (left) and *E*_*x*_ (right) at *y* = 0 for an 800 nm pulse of *s* = 50 (duration *τ*_0_ = 4.25 fs) at an NA of 0.8 with angular dispersion parameterized by *d* = *τ*_0_/2 at *z* = {0, 0.5, 1.0, 1.5} μm (from top to bottom).

Beyond the focal plane, the behavior at finite *z* is even more complex. Results are shown in Figure 6 at four *z* positions for the same beam as in Figure 5 at a fixed value of angular dispersion of *d* = *τ*_0_/2. The first apparent property is the extremely abrupt diffraction of a beam initially localized on the submicron level – on propagation by only 1.5 μm, the beam is roughly 10 times larger in extent. Another clear phenomenon is the appearance of the counter-propagating component of the field – at *z* = 0 the two components precisely overlap for a brief moment, but away from *z* = 0 they begin to separate in time. Last, we also clearly see the large curvature of the phase and optical energy on propagation. However, all of these properties are not in any way due to the angular dispersion and would be seen on a beam without any STC focused to the same level.

The effects due to the angular dispersion at the focus can be seen clearly in the transverse field *E*_*x*_. What is a distorted but cleanly tilted beam at *z* = 0 quickly develops a space-time singularity on propagation. At the furthest point shown, *z* = 1.5 μm, the space-time vortex character of the transverse field is clear, seen in recent paraxial and lightly nonparaxial work for TM_01_ beams with spatial chirp [49]. However, at smaller values of *z* the vortex character is less clear, and there are transient interferences with the counter-propagating pulse. The development of this space-time vortex is a natural result of diffraction, where the angular dispersion at the focus transforms slowly into spatial chirp away from the focus (the beam at the focusing optic has only spatial chirp). However, as is expected, the manifestation of this in the highly nonparaxial case is much more complicated. This shows again how intuition from the paraxial scenario is only useful to a certain degree. Additionally, space-time effects at the focus are only localized in a very small longitudinal space due to not only the abrupt diffraction of such localized beams but also the effect on space-time properties of the beam that diffraction naturally has.

The behavior of the longitudinal field *E*_*z*_ on propagation is less clear, since it is even more localized and diffracts, therefore, even more rapidly than *E*_*z*_. We can see that the interferences with the counter-propagating pulse are apparently more severe at intermediate *z* values. At large *z* values, the longitudinal field is no longer localized purely on-axes, and becomes more interlinked with the diffraction of the transverse field, since in such a highly nonparaxial environment, they must be intimately linked. In any case, we can make the same conclusion regarding the longitudinal field component when there is angular dispersion in the focus, that the situation is more complex and deviates from our expectations from the paraxial scenario, and that any emergent effects are even more highly localized along *z*.

#### 3.5.2 Input with angular dispersion

**Figure 7:**
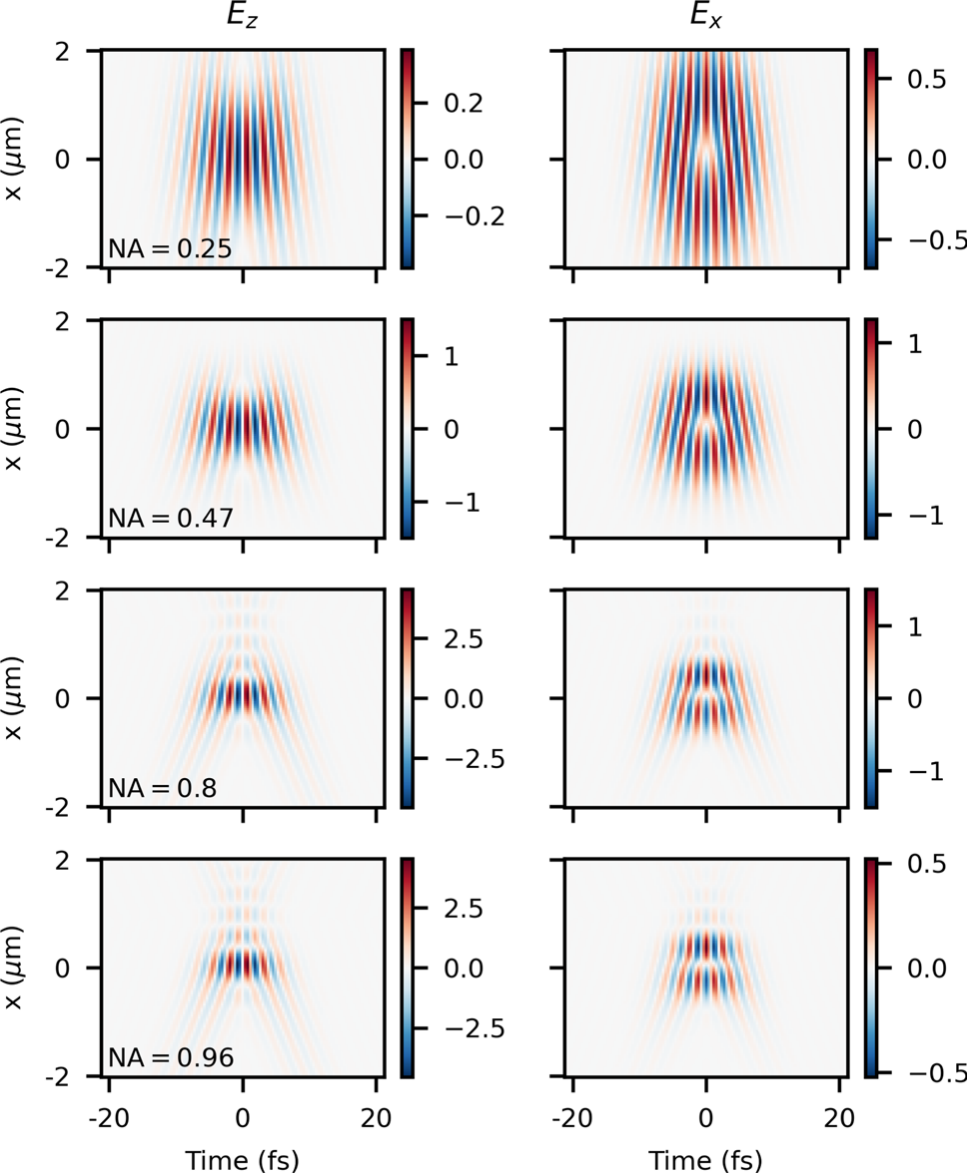
The electric field for both *E*_*z*_ (left) and *E*_*x*_ (right) at *z* = 0 for an 800 nm pulse of *s* = 50 (duration *τ*_0_ = 4.25 fs) at an NA of {0.25, 0.47, 0.8, 0.96} (from top to bottom) with spatial chirp parameterized by *τ*_*t*_ = *τ*_0_.

We can see in Figure 7 the fields obtained from the analytical model with spatial chirp at the focus (and thus angular dispersion as input) added according to *τ*_*t*_ = *τ*_0_ for a range of NA values. We see here, directly at the focus (*z* = 0), the same space-time vortex structure in the transverse field that we saw with angular dispersion away from the focus in Figure 6, and as was shown recently for the paraxial and mildly nonparaxial cases [49]. Now with spatial chirp at the focus, we can also clearly see the wavefront rotation in both *E*_*z*_ and *E*_*x*_, which is another indication of spatial chirp [50], [51].

#### 3.5.3 Input with chromatic curvature

**Figure 8:**
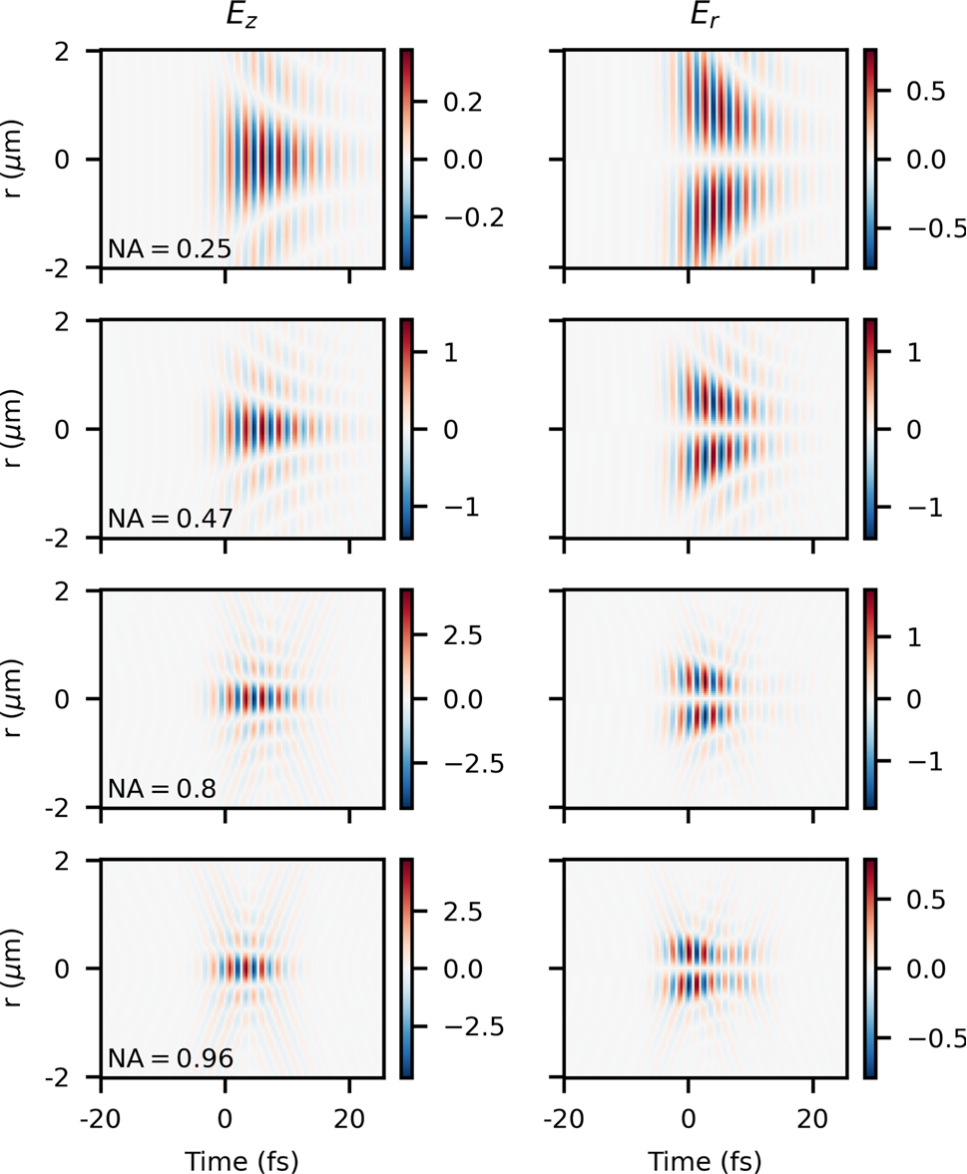
The electric field for both *E*_*z*_ (left) and *E*_*r*_ (right) at *z* = 0 for an 800 nm pulse of *s* = 50 (duration *τ*_0_ = 4.25 fs) at an NA of {0.25, 0.47, 0.8, 0.96} (from top to bottom) with longitudinal chromatism parameterized by *τ*_*p*_ = *τ*_0_.

Results of the analytical model with longitudinal chromatism (see field equations in Appendix C) are shown in Figure 8 at *z* = 0 and with one value of *τ*_*p*_ = *τ*_0_ for four different NA values. The first clear effects of the chromatism are the same as those seen in the paraxial case – an asymmetry in time and offset in time, which would be precisely reversed if the sign of the chromatism was opposite. Also, we note that the duration decreases from low NA to high NA, since the fixed value of *τ*_*p*_ is before focusing – when the beam is more tightly focused, the fixed value of chromatism results in less distribution of the frequencies along *z* and, therefore, less of a reduction of bandwidth at *z* = 0. This is the same conceptually as in the paraxial case. However, at the highest value of NA = 0.96, we see a warping of the space-time structure of only the transverse field *E*_*r*_, which is an effect only present with such tight focusing. We can learn more about the source of this by looking at the effects of propagation in such a highly nonparaxial situation.

**Figure 9:**
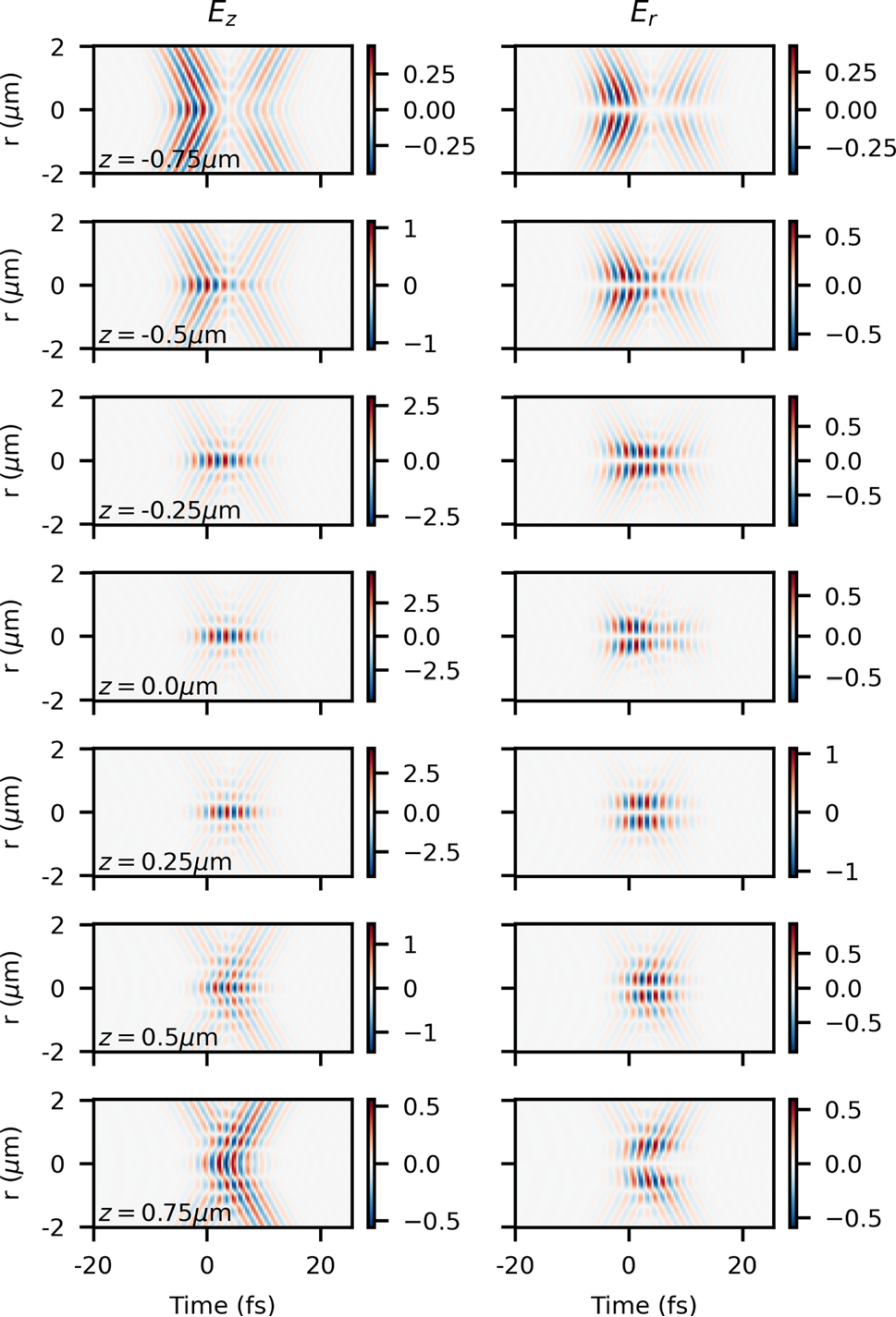
The electric field for both *E*_*z*_ (left) and *E*_*r*_ (right) for an 800 nm pulse of *s* = 50 (duration *τ*_0_ = 4.25 fs) at an NA of 0.96 with longitudinal chromatism parameterized by *τ*_*p*_ = *τ*_0_ shown at *z* = {−0.75, − 0.5, − 0.25, 0.0, 0.25, 0.5, 0.75} μm (from top to bottom).

More results of the analytical model with longitudinal chromatism are shown in Figure 9 with one value of *τ*_*p*_ = *τ*_0_ and NA = 0.96 for a range of *z* values, showing the effects upon propagation. We first notice an asymmetry not only in time but also in propagation along *z* – the best focus position if determined by the maximum intensity seems to be closer to *z* = 250 nm rather than at *z* = 0. Secondly, just like in other cases shown earlier, we see a significant interference with the counter-propagating solution at intermediate *z* values, only visible here at the tightest focusing level investigated. Finally, we notice a pseudo-nondiffracting profile present throughout propagation. We can gain intuition from the paraxial case with longitudinal chromatism, where the beam profile remains constant for some range of *z* (“extended Rayleigh range”) depending on the focusing level and magnitude of the chromatism [43]. However, in this highly nonparaxial case, the background level is high compared to the paraxial situation, and, naturally with tight focusing, this effect is extremely localized.

## 4 Adding chromatic aberrations to the integral model

A numerical tool called the StrattoCalculator has been developed to calculate electromagnetic fields at the focus of a parabolic reflector with high numerical aperture [52] using the Stratton–Chu integral formulation [30]. This code can accommodate for many input laser beams and complex mirror geometries [13]. Thus, this model can be used to confirm the results of the previous section. Refs. [41], [53] have recently shown first results of low-order STCs with such an integral model, but we will expand upon this work in this section by going to very high NA and by comparing with the analytical model introduced in previous sections.

One strength of this approach is that the description of spatio-temporal aspects of the input beam becomes simpler since the connection between the input beam and focal plane is no longer necessary as it is accomplished automatically within the diffraction integral calculations. Therefore, any spatio-temporal phase effect can be explicitly included in the input beam via frequency-dependent Zernike polynomials, for example.

The calculation with spatio-temporal couplings is performed in three steps:

1. A simple analytical collimated beam model described by the fields $E_{n}^{\text{beam}}\left(x\right)$ and $B_{n}^{\text{beam}}\left(x\right)$ is considered, with proper normalization using the Poynting theorem.
2. A set of transformations is applied to model the effect of optical components or to change its characteristics, which yield the incident field on the reflector surface $E_{n}^{\text{inc}}\left(x\right)$ and $B_{n}^{\text{inc}}(x).$

3. Stratton–Chu integrals are used to evaluate the corresponding focused field.

To connect with the analytical models presented in previous sections, the analytical beam model represents a collimated radially polarized beam (TM_01_ mode), whose fields are given explicitly by [54]:

(40) $$\begin{array}{ll} E_{n}^{\text{beam}}\left(x\right) & =\frac{2E_{0,n}}{k_{n}w_{i}^{2}}\;e^{-{\left(r/w_{i}\right)}^{2}+ik_{n}z}\\ & \;\times \left[r\;\hat{r}+\frac{2i}{k_{n}}\left(\frac{r^{2}}{w_{i}^{2}}-1\right)\;\hat{z}\right], \end{array}$$

(41) $$B_{n}^{\text{beam}}\left(x\right)=-\frac{E_{r,n}^{\text{beam}}\left(x\right)}{c}\;\hat{\phi },$$

where $r=\sqrt{x^{2}+y^{2}}$ is the radial distance, *k*_*n*_ is the wave number, and *w*_*i*_ is the Gaussian beam waist of the input beam. Other profiles could easily be modeled in future work. Then, the spatio-temporal couplings are applied via frequency-dependent Zernike polynomials, in the following way:

(42) $$E_{n}^{\text{inc}}\left(x\right)=E_{n}^{\text{beam}}\left(x\right){\text{e}}^{\text{i}S_{n}^{\text{Zernike}}\left(\rho ,\theta \right)},$$

(and similarly for the magnetic field), where the Zernike polynomial sum is given by

(43) $$S_{n}^{\text{Zernike}}\left(\rho ,\theta \right)=\underset{j\geq 0,m=-j,-j+2,\cdots ,j-2,j}{\sum }C_{j,n}^{m}Z_{j}^{m}\left(\rho ,\theta \right).$$

We can then set the value of coefficients $C_{j,n}^{m}$ to arbitrarily chosen values. In recent articles, a similar method to construct arbitrary space-frequency phases with Zernike polynomials in space and a Taylor expansion in frequency was used [55]. In the following sections, we will consider phases that are linear in frequency.

### 4.1 Input with angular dispersion

Angular dispersion on the input beam that results in the modeled spatial chirp, purely linear in frequency, can be described by a phase *ϕ* given by

(44) $$\phi \left(x,\omega \right)=\tau_{t}\delta \omega \frac{x}{w_{i}}\frac{\omega }{\omega_{0}}=\frac{\tau_{t}\omega \delta \omega }{2\omega_{0}}\left(Z_{1}^{1}\right),$$

where $Z_{1}^{1}$ is the Zernike polynomial representing *x*-tilt aberration and where the only nonzero coefficient is set to $C_{1,n}^{1}=\tau_{t}\omega_{n}\left(\omega_{n}-\omega_{0}\right)/2\omega_{0}$ , while the pupil is defined on {0, *w*_*i*_} (*ρ* = *r*/*w*_*i*_). If the pulse is relatively narrowband, Δ*ω* ≪ *ω*_0_, then the term *ω*/*ω*_0_ ∼ 1 and the phase is purely linear in frequency as well. When impinging on a focusing optic, as is simulated in the integral model, this phase will cause the frequencies to separate in space and form spatial chirp in the focus.

**Figure 10:**
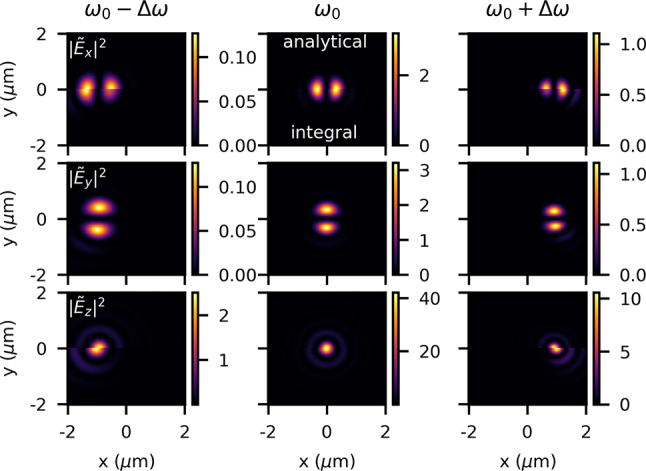
Spectral envelopes for all three electric field components ${\tilde{E}}_{x}$ , ${\tilde{E}}_{y}$ , and ${\tilde{E}}_{z}$ at *z* = 0 for an 800 nm pulse of *s* = 50 (duration *τ*_0_ = 2/Δ*ω* = 4.25 fs) at an NA of 0.8 with spatial chirp parameterized by *τ*_*t*_ = 3.6*τ*_0_ for the analytical model (upper half) and *τ*_*t*_ = 3*τ*_0_ for the integral model (bottom half). For reference, the corresponding wavelengths are 1,000 nm, 800 nm, and 667 nm.

To compare our two models, we look at the amplitude of the field components in frequency-space in Figure 10 with an NA of 0.8 (*w*_*i*_/*f* = 1, *k*_0_ *a* = 1.9). We use a larger value of spatial chirp of *τ*_*t*_ = 3*τ*_0_ for the integral model so that the effects are more clear. Firstly, to have the same offset in the analytical model, we needed to apply *τ*_*t*_ = 3.6*τ*_0_, meaning there is a mismatch between the simple scaling and the exact value from the integral method. This mismatch may be due to a number of factors, for example propagation from the input plane to the focusing parabola in the integral model, and we leave further exploration of the scaling to future work as detailed in Section 3.1. For these reasons, we don’t undertake any quantitative comparison in the case of spatial chirp at the focus.

We see in the analytical model, as expected, that the spatial chirp is manifested as only a spatial offset of the frequency components, in this case *ω*_0_ + Δ*ω* is centered at some positive *x* and *ω*_0_ − Δ*ω* is centered at some negative *x*, and all field components at a given frequency have the same offset. There are no other changes due to the spatial chirp. In the integral model, however, as expected from Ref. [42], not only is there a spatial offset, but the noncentral frequencies have an amplitude aberration causing an asymmetry along *x*. This also causes the longitudinal field to be pushed more outward, to conform to the asymmetry in the transverse fields.

One can also see in Figure 10 that the beam size depends on frequency, with higher frequencies having a smaller size. Albeit not necessarily expected, on examination of Eq. (3) (where *w*_0_ is still a relevant quantity for each individual field component) one can see that, with *k* = *ω*/*c*, there is a frequency-dependence of the beam size. If *k*_0_ *a* ≫ 1, then $w_{0}\propto 1/\sqrt{\omega }$ , but it is more complex when *k*_0_ *a* ∼ 1.

Interestingly, the frequency-dependence of the beam waist is slightly different in the case of the integral method, since the assumption in that case is that the input beam waist is frequency-independent. Looking at Eq. (7), if *w*_*i*_ is constant over frequency, then *a* will have a frequency-dependence ∝ 1/*ω*, and in turn so will the beam waist at the focus via Eq. (3) – the frequency-dependence is slightly stronger. In this case, the dependence is always the same, regardless of the focusing level.

In the end, however, the effects of such a frequency-dependent amplitude are not very significant in the case of spatial chirp at the focus, since all frequencies are still at their focus at *z* = 0, so it is only an amplitude effect. There are surely more interesting nuances in propagation, and the relative importance of these effects will increase with shorter pulse durations, but it is outside the scope of this work.

### 4.2 Input with chromatic curvature

A chromatic curvature on the input beam that results in the modeled longitudinal chromatism can be described by a phase *ϕ*

(45) $$\phi \left(r,\omega \right)=\tau_{p}\delta \omega \frac{\omega }{\omega_{0}}\frac{r^{2}}{w_{i}^{2}}=\frac{\tau_{p}\omega \delta \omega }{2\omega_{0}}\left(\frac{1}{\sqrt{3}}Z_{2}^{0}+Z_{0}^{0}\right),$$

where $Z_{0}^{0}$ is the piston aberration (which can essentially be ignored as a chromatic piston linear in frequency is only a shift in delay), $Z_{2}^{0}$ is the defocus aberration, i.e., $C_{2,n}^{0}=\tau_{p}\omega_{n}\left(\omega_{n}-\omega_{0}\right)/2\sqrt{3}\omega_{0}$ , and the pupil is defined on {0, *w*_*i*_} as before (*ρ* = *r*/*w*_*i*_). Once again, if Δ*ω* ≪ *ω*_0_, *ω*/*ω*_0_ ∼ 1 and the phase is purely linear in frequency. This frequency-dependent defocus aberration will cause the different frequencies to have different best focus positions, i.e., longitudinal chromatism.

**Figure 11:**
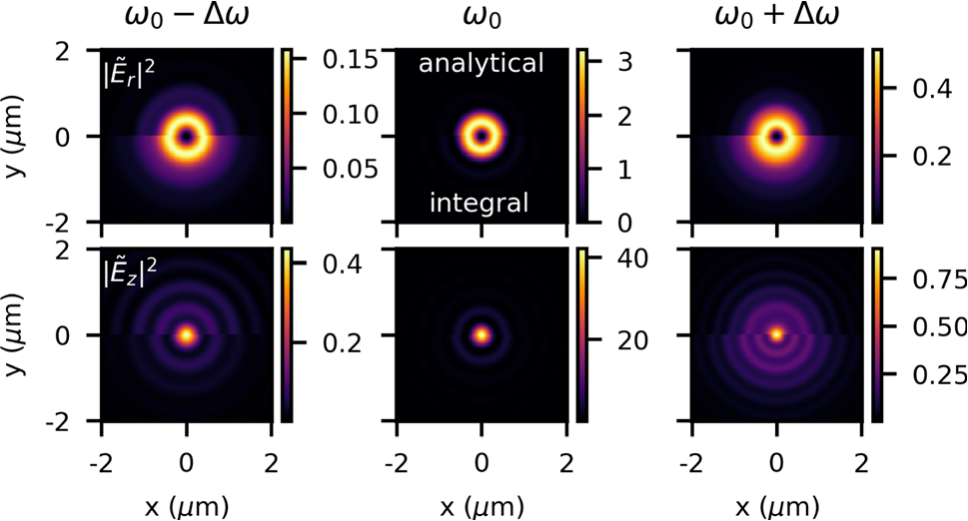
Spectral envelopes for all two electric field components ${\tilde{E}}_{r}$ and ${\tilde{E}}_{z}$ at *z* = 0 for an 800 nm pulse of *s* = 50 (duration *τ*_0_ = 2/Δ*ω* = 4.25 fs) at an NA of 0.8 with longitudinal chromatism parameterized by *τ*_*p*_ = *τ*_0_ for both the analytical (upper half) and integral models (bottom half).

We can see a comparison of the analytical and integral models in Figure 11. We notice that both techniques show the same general behavior – the noncentral frequencies are larger than the central frequency, since at *z* = 0, they are out of focus and have diffracted. However, we do see nuanced but significant differences.

The cause for these differences is linked to the frequency-dependent beam waist discussion from the previous section. Although in the analytical and integral models the longitudinal waist positions of each frequency are displaced by the same amount along *z* (i.e., at *z* = 0 the frequencies are the same distance out of focus), since the frequencies have slightly different beam sizes, they are out of focus by different magnitudes relative to their own beam waist. This means that between the analytical and integral models, the frequencies have diffracted at a different level; therefore, we see the different size and structure of the fields. This topic also helps us understand the warping in time and asymmetry in *z* that we saw in Figures 8 and 9.

In the case of LC at the focus in this section, we feel confident doing a quantitative comparison between the semi-analytical and integral techniques. We compare the intensities of the two cases, *I*_1_ and *I*_2_, that are normalized according to the L2 norm across the spatial profile (*∑I*_1,2_ = 1). Via the error calculation $\sum {\left(\sqrt{I_{1}}-\sqrt{I_{2}}\right)}^{2}/{\left(\sqrt{I_{1}}+\sqrt{I_{2}}\right)}^{2}$ , we can find that for *E*_*r*_ (*E*_*z*_), the error at the central frequency is 0.008 (0.032), but the error at *ω*_0_ + Δ*ω* is 0.023 (0.014), for example. Of course, the more important aspect is that the space-time-polarization shape is different, which cannot be quantified in one number, and only for a given application can one determine which model is appropriate.

### 4.3 Higher-order frequency-dependent aberrations

**Figure 12:**
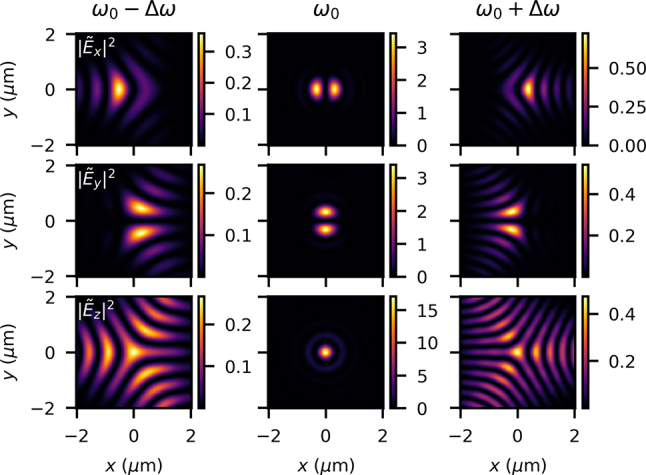
Spectral envelopes for all three electric field components ${\tilde{E}}_{x}$ , ${\tilde{E}}_{y}$ , and ${\tilde{E}}_{z}$ at *z* = 0 for an 800 nm pulse of *s* = 50 (duration *τ*_0_ = 2/Δ*ω* = 4.25 fs) at an NA of 0.8 with chromatic trefoil parameterized by *τ*_*o*_ = 2*τ*_0_.

Our advanced integral model allows for the investigation of even higher-order aberrations and combination thereof, in this highly nonparaxial situation. Chromatic astigmatism has recently been described for the paraxial case [56], but the integral model can easily handle higher orders. Chromatic oblique trefoil is an example of such an aberration, since it is a frequency-dependent Zernike polynomial of higher order than tilt or defocus studied in previous sections, or astigmatism studied in past work. Figure 12 provides an example of the higher-order aberration described by a phase *ϕ*.

(46) $$\phi \left(r,\omega \right)=\frac{\tau_{o}\omega \delta \omega }{2\omega_{0}}\left(\frac{1}{\sqrt{8}}Z_{3}^{3}\right).$$

## 5 Conclusions

In this work, we have investigated how relatively simple space-time effects on the input collimated beam will manifest at the focus of optics with arbitrary level of focusing. In the paraxial case, which has been expounded upon significantly in past work, the transfer of STCs from the input beam to the focal region is well understood, has straightforward scalings, and importantly does not involve any coupling between polarization components [57]. However, as we have shown in this manuscript, when the focusing becomes nonparaxial, the geometry of the focusing situation becomes very important, the scaling of the STC at the focus becomes more nuanced, and we must include the full vectorial nature of diffraction to get the most complete results.

We performed our analysis, with both the complex source-point analytical model and the Stratton–Chu integral model, exclusively on the lowest-order radially polarized mode (TM_01_). This was because it is the simplest mathematically and is known for creating a tight focus. However, since the analytical model was constructed on vector potentials, our techniques apply equally well for other input beams as long as they are the lowest-order spatial mode, which only require a different choice of the orientation of the vector potential(s). The integral model is also fully capable to handle an arbitrary input intensity and polarization distribution, and the space-time modeling via frequency-dependent Zernike polynomials would be applied in the same fashion as presented here.

To summarize our results concisely, nonparaxial focusing means that the standard modeling and intuition for space-time couplings no longer applies, and that both the scaling and the vector components in the focus are more complex than in paraxial focusing. The analytical model with our corrections provides quite accurate results, but the integral model captures additional nuances like focused spatial profile distortions and propagation effects between the input plane and the parabola. Only each specific application can determine whether the analytical model or integral model is necessary, but in this work, we have built the tools and shown the results.

Although there are some methods to measure the fields in such tight focusing geometries – either for linearly polarized light [58], [59], for measuring specifically the longitudinal field [60], [61], [62], [63], [64], [65], [66], or for arbitrary vectorial distributions [67], [68] – this is still a focus of current research, and there is so-far no demonstration of space-time measurements on ultrashort pulses in such geometries. This was a topic of discussion in a recent perspective of space-time measurements [69], and it underscores the importance of this current work – the space-time fields of an ultrashort pulse within a highly nonparaxial environment are both complicated to describe and difficult to measure, so modeling is of crucial importance.

There is important future work along this same direction, for example modeling different types of beams or even higher-order beams, diving deeper into the analysis of properties of the beams or behavior along propagation, or addressing other nuances like the modeling of frequency-dependence of the beam waist or other behaviors that become important at even shorter pulse durations than presented here. It may also be possible to describe some specific couplings or beams fully analytically in time, by solving the Fourier transform of a specific equation presented here. But in this work, we have built a strong foundation for modeling some of the most extreme and complex electromagnetic wave configurations and hopefully enabling their use for nanophotonic or high-field applications.

## Appendix

### A Fields in time

If $\Pi_{e}=\Psi \hat{z}$ , the other two field components can be calculated as such

(47)
$$E_{r}=\frac{\partial^{2}\Psi }{\partial z\partial r},$$

(48)
$$B_{\theta }=-\frac{1}{c^{2}}\frac{\partial^{2}\Psi }{\partial t\partial r}.$$

The solutions are

(49)
$$\begin{array}{ll} E_{r} & =\frac{A_{0}r\left(z+ia\right)}{{\tilde{R}}^{3}}\left[\frac{3}{{\tilde{R}}^{2}}\left(f\left({\tilde{t}}_{+}\right)-f\left({\tilde{t}}_{-}\right)\right)\right.\\ & \;-\left.\frac{3}{c\tilde{R}}\left(f^{\prime }\left({\tilde{t}}_{+}\right)+f^{\prime }\left({\tilde{t}}_{-}\right)\right)+\frac{1}{c^{2}}\left(f^{\prime \prime }\left({\tilde{t}}_{+}\right)-f^{\prime \prime }\left({\tilde{t}}_{-}\right)\right)\right], \end{array}$$

(50)
$$B_{\theta }=\frac{A_{0}r}{c{\tilde{R}}^{2}}\left[\frac{1}{\tilde{R}}\left(f^{\prime }\left({\tilde{t}}_{+}\right)-f^{\prime }\left({\tilde{t}}_{-}\right)\right)-\frac{1}{c}\left(f^{\prime \prime }\left({\tilde{t}}_{+}\right)+f^{\prime \prime }\left({\tilde{t}}_{-}\right)\right)\right].$$

### B Fields in frequency space

If ${\tilde{\Pi }}_{e}=\tilde{\Psi }\hat{z}$ and there are no STCs, *E*_*r*_ can be calculated the same way as was done in time but acting on $\tilde{\Psi }$ , and *B*_*θ*_ can be calculated as such

(51)
$${\tilde{B}}_{\theta }=-\frac{i\omega }{c^{2}}\frac{\partial \tilde{\Psi }}{\partial r},$$

with the solutions as follows

(52)
$$\begin{array}{ll} {\tilde{E}}_{r} & ={\tilde{A}}_{0}F\left(\omega \right)e^{-ka}\left(\frac{r\left(z+ia\right)}{{\tilde{R}}^{3}}\right)\\ & \;\times \left[\sin\left(k\tilde{R}\right)\left(\frac{3}{{\tilde{R}}^{2}}-k^{2}\right)-\cos\left(k\tilde{R}\right)\left(\frac{3k}{\tilde{R}}\right)\right], \end{array}$$

(53)
$$\begin{array}{ll} \;{\tilde{B}}_{\theta } & ={\tilde{A}}_{0}F\left(\omega \right)e^{-ka}\left(\frac{ikr}{c{\tilde{R}}^{2}}\right)\times \left[\sin\left(k\tilde{R}\right)\left(\frac{1}{\tilde{R}}\right)-k\cos\left(k\tilde{R}\right)\right]. \end{array}$$

### C Fields with STCs

If there is only a spatial chirp and longitudinal chromatism (*θ*(*ω*) = 0), then ${\tilde{R}}_{\text{STC}}=\sqrt{r_{\text{STC}}^{2}+{\left(z-z_{0}\left(\omega \right)+ia\right)}^{2}}$ and we can calculate the following field components based on the Cartesian derivatives

(54)
$${\tilde{E}}_{z}=-\left(\frac{\partial^{2}\tilde{\Psi }}{\partial x^{2}}+\frac{\partial^{2}\tilde{\Psi }}{\partial y^{2}}\right),$$

(55)
$${\tilde{E}}_{x}=\frac{\partial^{2}\tilde{\Psi }}{\partial z\partial x},$$

such that

(56)
$$\begin{array}{ll} {\tilde{E}}_{z} & ={\tilde{A}}_{0}F\left(\omega \right)e^{-ka\left(\omega \right)}e^{-ikz_{0}\left(\omega \right)}\\ & \;\times \left[\sin\left(k{\tilde{R}}_{\text{STC}}\right)\left(\frac{2+k^{2}r_{\text{STC}}^{2}}{{\tilde{R}}_{\text{STC}}^{3}}-\frac{3r_{\text{STC}}^{2}}{{\tilde{R}}_{\text{STC}}^{5}}\right)\right.\\ & \;+\left.\cos\left(k{\tilde{R}}_{\text{STC}}\right)\left(\frac{3kr_{\text{STC}}^{2}}{{\tilde{R}}_{\text{STC}}^{4}}-\frac{2k}{{\tilde{R}}_{\text{STC}}^{2}}\right)\right], \end{array}$$

(57)
$$\begin{array}{ll} \;{\tilde{E}}_{x} & ={\tilde{A}}_{0}F\left(\omega \right)e^{-ka\left(\omega \right)}e^{-ikz_{0}\left(\omega \right)}\\ & \;\times \left(\frac{\left[x-x_{0}\left(\omega \right)\right]\left[z-z_{0}\left(\omega \right)+ia\right]}{{\tilde{R}}_{\text{STC}}^{3}}\right)\\ & \;\times \left[\sin\left(k{\tilde{R}}_{\text{STC}}\right)\left(\frac{3}{{\tilde{R}}_{\text{STC}}^{2}}-k^{2}\right)\right.\\ & \;-\left.\cos\left(k{\tilde{R}}_{\text{STC}}\right)\left(\frac{3k}{{\tilde{R}}_{\text{STC}}}\right)\right], \end{array}$$

where only *E*_*x*_ and *E*_*z*_ are shown to be concise.
